# The Role of Artificial Intelligence in Cardiovascular Imaging: State of the Art Review

**DOI:** 10.3389/fcvm.2020.618849

**Published:** 2020-12-23

**Authors:** Karthik Seetharam, Daniel Brito, Peter D. Farjo, Partho P. Sengupta

**Affiliations:** Department of Cardiology, West Virginia University Medicine Heart & Vascular Institute, Morgantown, WV, United States

**Keywords:** machine learning, automation, cardiovascular imaging, precision medicine, advanced imaging

## Abstract

In this current digital landscape, artificial intelligence (AI) has established itself as a powerful tool in the commercial industry and is an evolving technology in healthcare. Cutting-edge imaging modalities outputting multi-dimensional data are becoming increasingly complex. In this era of data explosion, the field of cardiovascular imaging is undergoing a paradigm shift toward machine learning (ML) driven platforms. These diverse algorithms can seamlessly analyze information and automate a range of tasks. In this review article, we explore the role of ML in the field of cardiovascular imaging.

## Introduction

As technology continues to evolve at a rapid pace, each and every significant innovation has a phenomenal capability of transforming various aspects of society (1, 2). In this era of miniaturized devices and smartphone applications, continuous streams of individualized information have become the standard of life (3, 4). Similarly, the adoption of artificial intelligence (AI) in medicine has far-reaching potential, especially in the field of cardiovascular imaging (2, 5, 6). With a vast array of imaging modalities at our disposal, these approaches provide limitless information regarding cardiac structure and function (7). In parallel to the technological revolution, imaging approaches also continue to grow significantly (5, 8). Novel parameters are added to existing techniques, providing additional information regarding the cardiac function (7, 9, 10). However, are more data points beneficial if they cannot be used in routine clinical practice? (11). Information necessary to medical management needs to be prioritized first rather than having a cacophony of data points.

The utility of AI in cardiovascular imaging bridges the gap between new technology, big data, and the clinical provider (12–14). Machine learning (ML), a branch of AI, is especially pertinent in cardiovascular imaging as it can analyze large amounts of information in a multitude of approaches (1, 15, 16). ML can connect information from a variety of interfaces and present it in a meaningful manner for the practitioner (13, 14). Also, it can automate several measurements in various imaging modalities (17, 18). The growth of AI will facilitate the progression of precision medicine. In this review article, we assess the role of AI and ML in cardiovascular imaging.

## Evolution of Big Data

Imaging modalities permit the visual assessment of cardiac function and detect underlying cardiac pathology. A single scan produces an abundance of clinical and operational data (19). For example, a single echocardiogram can produce roughly 2 gigabytes of data. Since millions of patients undergo echocardiography annually, this translates to petabytes of information being collected. With the emergence of higher processing capabilities of computer processing units (CPU) and cloud infrastructures, the newer system can process a plethora of data in real-time (5, 20). Imaging data can be quite complex and present in varying dimensions (two or three or 4 dimensions) and formats which include digital imaging and communications in medicine (DICOM), moving picture experts Group (MPEG), and joint photographic experts group (JPEG) (19). This results in exceedingly high dimensionality of data and predisposes to significant difficulties in clinical practice (21).

Big data is heavily utilized in clinical research (22). Many academic centers invest vast resources in generating and large data sets for various research endeavors. The findings from large data sets are generally applicable to vast portions of the population. They provide more consistent and reliable findings than smaller or single institutional studies. In the near future, big data will incorporate genetic or molecular parameters for the patient or pan-omic data sets (22, 23).

## Emerging Significance of AI

As stated earlier, data is becoming increasingly complex with rapidly advancing changes in technology (18). Big data with countless, non-linear associations will exceed the capabilities of existing frequentist or Bayesian statistical approaches (23, 24). Although they are the current gold standard in current research, this may not hold true in the foreseeable future.

In contrast, ML and AI are far more dynamic in nature (14). With this transition to big data, ML algorithms will play a pivotal role in the days to come. As the size and complexity of data increases, the performance of ML increases proportionally (5, 25). ML frameworks can further expand our knowledge regarding different cardiac pathology. It can connect information from a variety of different interfaces.

## Types of Machine Learning

ML is an umbrella term that refers to a collection of various analytical algorithms [(5); Table 1]. It can be broadly classified into supervised learning, unsupervised learning, semi-supervised learning, and reinforcement learning (26). Among these, supervised and unsupervised learning are frequently used in clinical research (27). Supervised learning operates within domains of labels or annotations within a dataset (18, 28). Whereas, unsupervised learning looks purely at data points independent form labels and is considered agnostic (29, 30). Semi-supervised learning contains properties of both supervised and unsupervised learning (12). The last among these ML frameworks is reinforcement learning. Reinforcement learning is similar to human psychology, utilizes certain reward criteria for the algorithm to perform functions within a dataset (22). It is yet to gain significant traction in the field of cardiovascular imaging.

**Table 1:** A table describing different types of machine learning with various examples.

**Types of machine learning**	**Function**	**Examples**
Supervised learning (12)	The dataset has labels and outcomes, infers from data for prediction purposes	Encompasses logistic regression, ridge regression, elastic net regression, Bayesian network, artificial neural network
Unsupervised learning (12)	The dataset contains no labels, detects pivotal relationships	This contains hierarchical clustering, k-means clustering, principal component analysis
Semi-supervised learning (12)	A combination of supervised and unsupervised learning	Frequently used in image and speech recognition
Re-enforcement learning (12)	Utilizes reward function to execute tasks	Commonly seen in medical imaging, analytics, and prescription selection

## Rising Relevance of Deep Learning

Among ML algorithms, deep learning has the most potential in innovation and discovery (24, 31). It is becoming increasingly popular due to significant strides in cloud infrastructures and augmented computing prowess (12). Deep learning is the foundation for cutting-edge technology like voice recognition software such as Siri or Alex and self-driving cars (1, 32). In comparison to other ML frameworks, deep learning performs significantly better with larger and complex datasets. The architecture of deep learning is similar to the neuronal structure present within humans (33, 34). Arranged in a series of layers, information is processed from preceding and successive layers in an intricate manner to extrapolate outcomes present within vast data matrices (25). Other algorithms require significant training to obtain satisfactory results. However, the accuracy of deep learning can be easily improved by elevating the network capacity or augmenting the training dataset. It requires less domain knowledge to execute a function.

There are also several subtypes present in deep learning. One of the most commonly used deep learning frameworks is a convolutional neural network (CNN) (31). It contains a convolutional component responsible for feature extraction and has a fully connected enabling classification. In fully connected networks (FNN), every unit in any layer is linked to every unit in prior and succeeding layers (31). Recurrent neural networks (RNN) employ feedback loops to comprehend various inputs (31).

## Role of AI in Echocardiography

**Figure 1:**
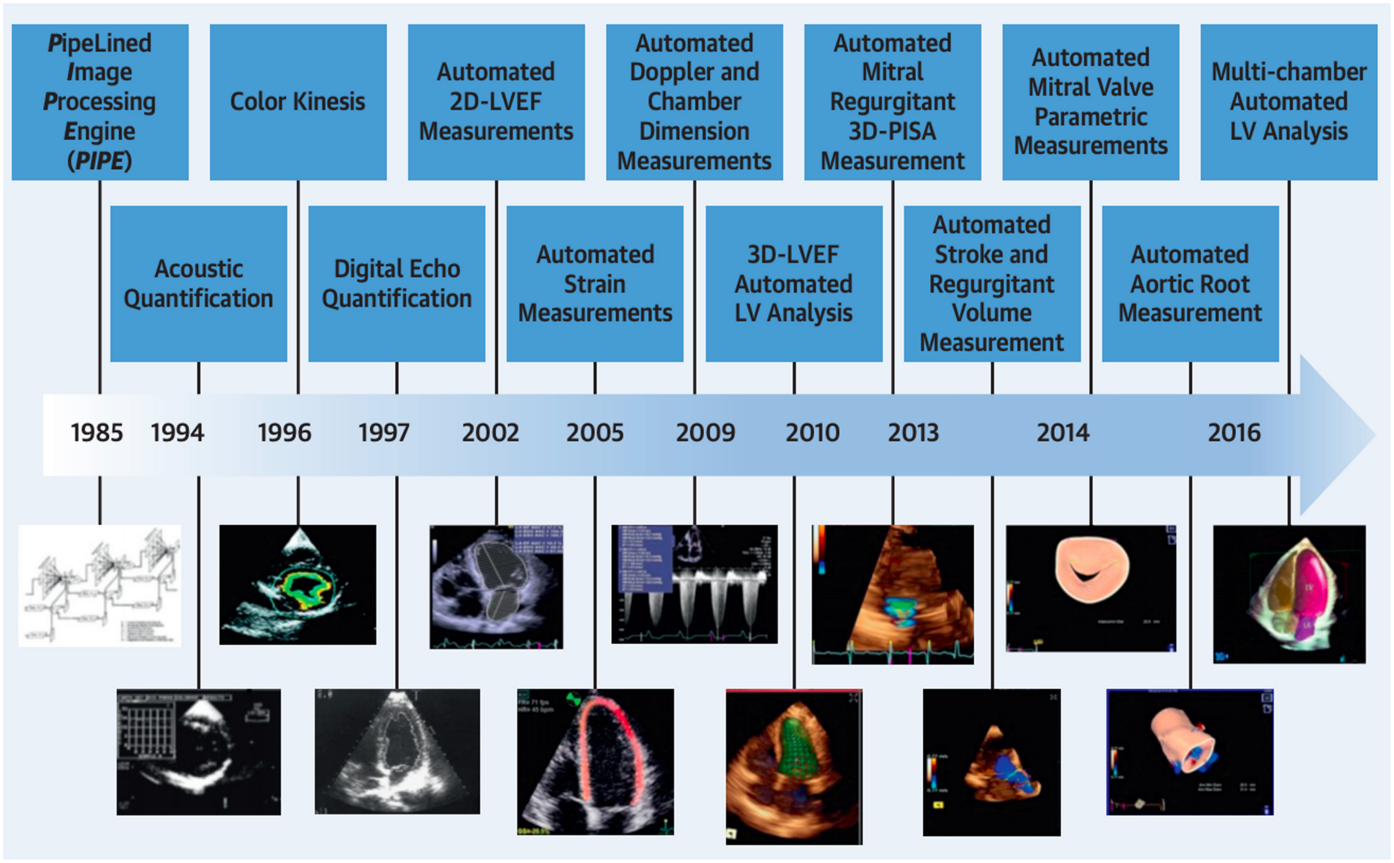
Temporal progression in automated quantification in echocardiography. Adapted from Nolan et al. (10). Permission obtained from the publisher.

Echocardiography is the most widely used imaging technique in cardiac care (10). It plays an indispensable role in assessing cardiac function and it identifies various cardiac pathology (10). Over the last 33 years, significant progression in echocardiographic automated quantification has occurred (Figure 1).

ML algorithms can make new echocardiographic technology clinically relevant. With the emergence of cardiac strain, it can evaluate the cardiac function beyond the ejection fraction. Ejection fraction is hailed as the gold standard metric for cardiac assessment. Although strain can fundamentally alter clinical care, it has not occurred due to the cumbersome and time-consuming nature of the approach (35). The integration of ML algorithms can streamline the clinical workflow by automating numerous calculations (9).

Samad et al. utilized clinical and echocardiographic variables using a random forest model which achieved superior prediction accuracy (all AUC >0.82) over common clinical risk scores (AUC = 0.69–0.79) and logistic regression models (*p* < 0.001) in 171, 510 patients for predicting all-cause mortality (36). Zhang et al. trained a (CNN) which successfully identified views (96% for parasternal long axis or 84% accuracy overall) with image segmentation accuracy reaching 72–90% (37). Sanchez-Martinez et al. assessed velocity patterns to differentiate heart failure preserved ejection fraction (HFpEF) from healthy patients with encouraging results (κ, 72.6%; 95% confidence interval, 58.1–87.0) (38). Similarly, Tabassian et al., classified phenotypes of HFpEF patients with symptomatology using strain parameters (asymptomatic vs. symptomatic; AUC = 0.89; accuracy = 85%; sensitivity = 86%, specificity = 82%) (39). Unsupervised clustering models in HFpEF patients have successfully predicted hospitalization risk, exercise intolerance, and LV filling pressure (40–43). Lancaster et al. applied clustering ML model which isolated diastolic dysfunction in 559 of 866 patients with 2 distinct groups, revealed moderate agreement with conventional classification (kappa= 0.41, *p* < 0.001) (44). Asch examined ML automated echocardiographic quantification of left ventricular ejection fraction (LVEF), there was an excellent agreement with reference values (*r* = 0.95) (45). Benjamin et al. applied deep learning which showed lung Doppler signals (LDS) predicted echocardiographic E/e' measurements [*r* = 0.67 (admission) and 0.83 (discharge), *p* < 0.0001] in 99 acute HF patients and lower event-free survival in high predicted- E/e' group HF patients with reduced EF (*P* = 0.0247) (46). Kusunose et al. showed deep learning was better than residents in detecting wall motion abnormalities (WMA) (AUC 0.99 vs. 0.90, *p* = 0.002) and WMA territories (AUC = 0.97 vs. 0.83, *p* = 0.003), the ML architecture had a similar performance to experienced cardiologists (47). Similarly, Kusononse used deep learning to demonstrate similar ML derived LVEF with validation group (*r* = 0.82 ± 0.02, *p* < 0.001) (48). Donal utilized a random forest model to assess response to cardiac resynchronization therapy, best performance was obtained with strain measurements (AUC of 0.686) and QRS duration (AUC of 0.668) (49).

**Figure 2:**
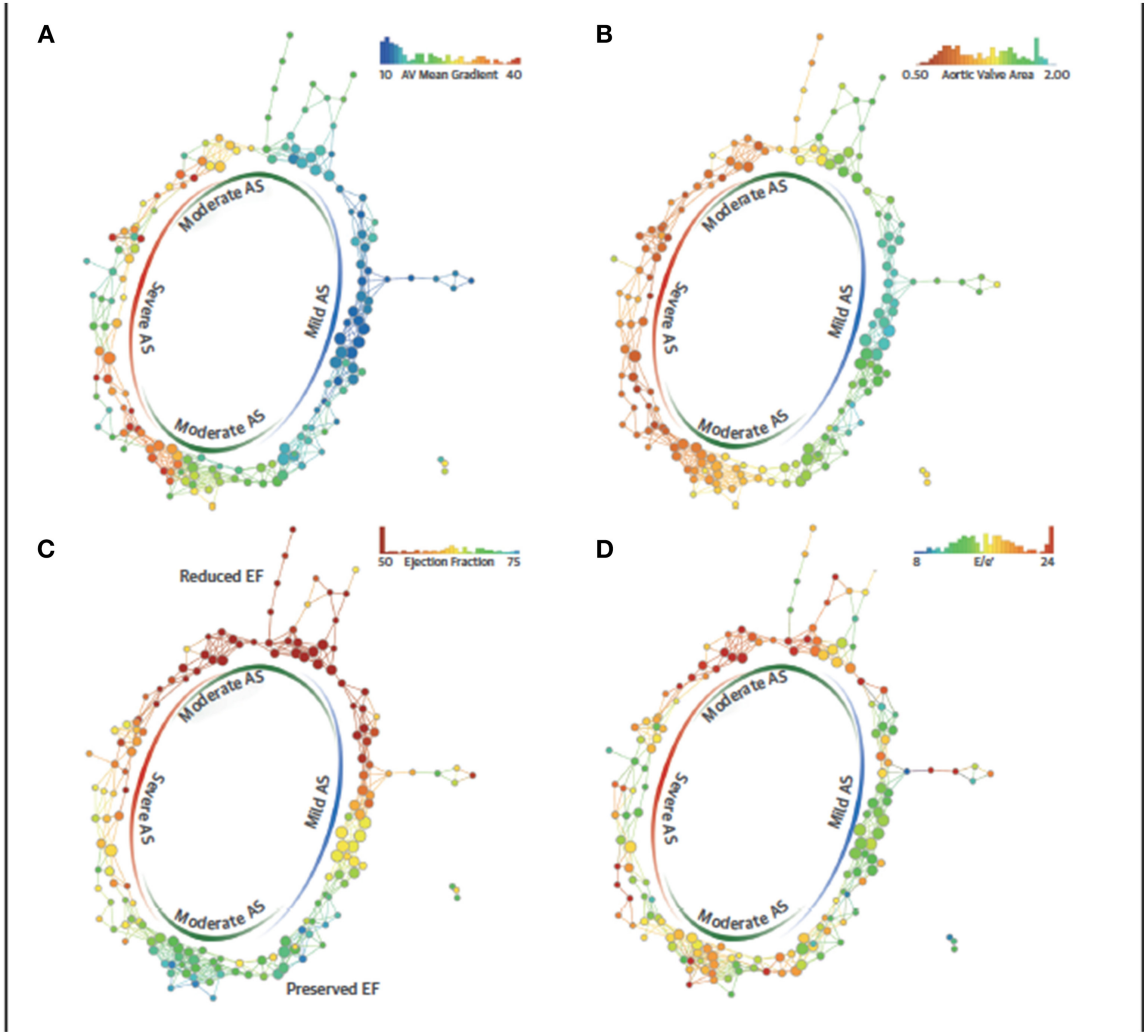
Topological data analysis loop in aortic stenosis. The similarity network draws aggregation of patients with severe and mild AS in 2 opposite ends and moderate AS in the top and bottom arms of the loop. The red color represents the abnormal values, whereas blue represents the normal or minor malady in the disease space. **(A)** Spectrum of mean gradient in humans identified in the network of disease space. High gradients aggregated on the left side, whereas the low gradients were on the right side with moderate gradients in the middle. **(B)** Distribution of valve area on the network, **(C)** patient similarity network revealing preserved and reduced ejection fraction (EFs) in the patients with aortic stenosis (AS). The top arm clustered patients with the reduced EF, whereas the bottom arm described the patients with preserved EF. The red nodes consists of patients with EFs of ≤50%, whereas the blue nodes consists of patients with EFs of ≥65%. **(D)** Distribution of E/e' as a feature of diastolic dysfunction revealing the pattern of E/e' around moderate and severe AS. TDA, topological data analysis. Adapted from Casaclang-Verzosa et al. (51). Permission obtained from the publisher.

Topological data analysis (TDA) is a form of unsupervised learning which uses clustering to create network and shape (50). Casaclang-Verzosa et al. employed TDA to discern precise LV phenotypic recognition in severe aortic stenosis (AS) patients, it formed a loop (Figure 2) of mild and severe aortic stenosis on the right and left side (*p* < 0.001) which was linked by moderate AS on top and bottom sides with reduced and preserved ejection fraction (*p* < 0.0001) (51). Other centers have used TDA in AS patients similarly, heart failure patients, and for predicting major adverse cardiovascular events (MACE) in large cohorts (52–54).

Several cardiovascular diseases can affect the cardiac valve. Previously surgical techniques were the gold standard techniques in valve intervention. With the advent of transcatheter approaches, there has been a paradigm shift in these approaches. The application of ML algorithms can help in the gradient and assessment of valvular heart disease. Also, they can help in therapeutic planning. Currently, is it still in the early stages. Costa et al. utilized deep learning to segment mitral valves in PLAX and apical 4 chamber views (55). Grady examined the role of ML for automating the proximal isovelocity surface area (PISA) assessment on echocardiography, it had excellent correlation with findings on magnetic resonance imaging (56). Wang et al. applied ML for evaluating mitral inflow and aortic outflow (57). Abdul Ghaffar et al. evaluated the role of semi-supervised learning for phenogrouping based risk assessment in transcatheter aortic valve replacement (TAVR) (58). Group 5 was associated with significant in-hospital cardiovascular mortality (OR 3.5, *p* = 0.001).

## Role of AI in Computed Tomography

In recent years, computed tomography (CT) has emerged as a prominent technique in the field of cardiovascular imaging (6, 59, 60), in part due to negative predictive value (61). The evolution of scanner technology has led to drastic improvements in spatial and temporal resolution (60, 62). ML algorithms can automate and expedite many processes which will expand the frontiers of cardiac CT (5).

**Figure 3:**
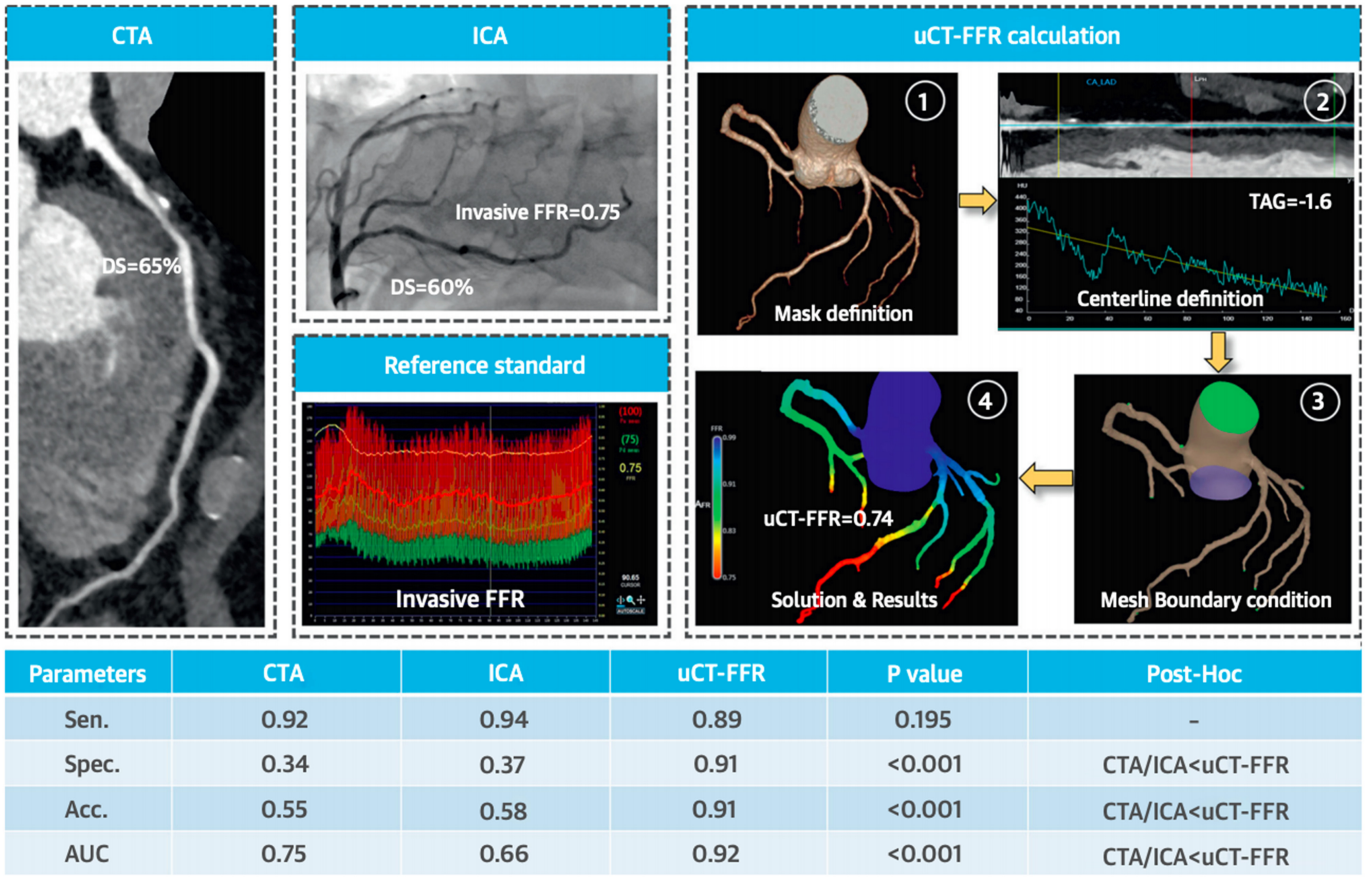
Functional significance of proximal left anterior descending artery stenosis. Adapted from Tang et al. (65). Permission obtained from the publisher.

CT fractional flow reserve (CT- FFR) is arising as a non-invasive alternative in diagnosing chest pain. Though in the early stages of clinical implementation, it is one of the few methods to provide an anatomical and functional assessment. ML algorithms can compute FFR without computational fluid dynamics and provide additional prognostic information (6). Zhou et al. investigated the role of CT fractional flow reserve (CT FFR) for predicting myocardial bridge formation by multiple ML algorithms, ML selected features had higher AUC (0.75 ± 0.04) than clinical features (0.53 ± 0.09, *p* < 0.0001), morphological features (0.59 ± 0.06, *p* = 0.0025), and CT- FFR features (0.62 ± 0.06, *p* = 0.0127) (63). Coenen et al. compared CT- FFR with computational fluid dynamics and ML-derived CT- FFR for detecting coronary artery disease (CAD) through deep learning, there was an excellent correlation between both techniques (*R* = 0.997) (64). Tang et al. noted that a novel on-site computational fluid dynamics-based CT FFR was better than CTA and invasive angiography in detecting lesion-specific ischemia, especially in intermediate lesions (*p* < 0.001 for all) [(65); Figure 3].

The presence of extensive coronary calcium (CAC) is the predominant issue in CAD evaluation (62). Furthermore, CAC can lead to overestimation of coronary vessel stenosis (66, 67). Al'Aref et al. utilized an ML model integrating clinical factors in conjunction with calcium score in the CONFIRM registry for predicting coronary artery disease in 35, 281 patients with CTA (68). They demonstrated superior AUC for ML and (CAC) (0.881) to ML alone (0.773), coronary calcium (0.886), and updated Diamond- Forrester Score (0.682). Similarly, Tesche et al. showed ML CT fractional flow reserve (FFR) was better than CTA alone in CAC, there was a significant difference in performance as Agatston scores increased (High scores- *p* = 0.04, low to intermediate scores- *p* < 0.001) (69). Kay et al. applied ML algorithms and radiomics to detect phenotypic information about high risk left ventricular hypertrophy (LVH) in CT with coronary artery calcium (CAC) scoring, these algorithmic models were highly effective in LVH detection (70). Hou et al. utilized supervised learning to calculate pretest probability in 6274 patients from CTA, the ML model had significantly higher discrimination for obstructive CAD than modified Diamond- Forester score and CAD consortium score (0.801 vs. 673 vs. 669, *p* < 0.001) (71).

ML architectures have been used in a variety of different situations in CT. Baskaran et al. applied deep learning which verified with manual annotation for left ventricular volume (*r* = 0.98), right ventricular volume (*r* = 0.97), and atrial volumes in CT angiography (CTA) (*P* < 0.05) in 166 patients (72). Oikonomou et al. utilized a random forest model to predict cardiac risk from the radiomic profile of coronary perivascular adipose tissue (PVAT) in CTA, the fat radiomic profile (FRP) were able to augment MACE prediction beyond conventional risk stratification scores (C statistic −0.126, *p* < 0.001) (73). Beecy et al. explored the role in acute ischemic stroke identification on CT, the AUC for the ML model was 0.91 for automated diagnosis of infarction and had a 93% diagnostic accuracy with expert physician interpretation (74). Al'Aref et al. investigated the potential of supervised learning in CTA to identify culprit lesion precursors from acute coronary syndrome patients, the ML model displayed higher AUC for distinguishing precursors than multiple other models (0.774 vs. 0.599 vs. 0.532 vs. 0.672, *p* < 0.01) (75). Eisenberg used deep learning to show epicardial adipose tissue (EAT) volume (HR 1.35, *p* < 0.01) predicted MACE on CT, while attenuation (0.83, *p* = 0.01) had an inverse relationship (76).

## Role of AI in Nuclear Cardiology

Single-photon emission computed tomography (SPECT) myocardial perfusion imaging (MPI) is the cardinal test in nuclear cardiology, plays a paramount role in the assessment of obstructive CAD (17). SPECT is predominantly used to evaluate myocardial perfusion and to identify possible perfusion defects either during rest or stress imaging indicating underlying ischemia (17). There are significant disparities in the diagnostic performance of SPECT attributed to many aspects that can be addressed by ML (77).

Betancur demonstrated deep learning was superior to total perfusion deficit (TPD) in MPI for CAD prediction (78, 79). With unsupervised learning, Betancur et al. exhibited higher (MACE) compared to expert readers, automated total perfusion deficit (TPD), and automated ischemic perfusion deficit in SPECT MPI and clinical factors for 2619 patients (AUC: 0.81 vs. 0.65 vs. 0.73 vs. 0.71, *p* < 0.01 for all) (80). Hu et al. explored the role of ML networks in automatic rest scan cancellation and prognostic safety, patients selected for rest scan cancellation had lower annualized MACE rates than the physician or clinical selection rules (all, *P* < 0.0001) (81). Otaki et al. compared ML with visual reading for predicting MACE in 19,495 patients, it enabled more precise risk stratification than visual analysis (82). Juarez-Orozco et al. assessed the role of deep learning in 1,185 patients for polar maps in ischemia by positron emission tomography (PET), deep learning had an AUC of 0.90 ± 0.02 and outperformed all comparator models (all pairwise *p* < 0.01) (83). Hu et al. investigated the ML algorithm to per- vessel prediction of early coronary revascularization within 90 days of SPECT MPI, they found ML AUC was superior to regional stress TPD, combined- view TPD, and ischemic TPD (0.79 vs. 0.71 vs. 0.72, *P* < 0.001) (84).

## Role of AI in Cardiac Magnetic Resonance Imaging

Over the last several years, cardiac magnetic resonance imaging (CMR) has emerged as an indispensable tool in the field of cardiovascular imaging (12). Substantial strides in the technological front have enhanced the capability of CMR for risk stratification and diagnosis. CMR is heralded as the gold standard for non-invasive assessment of the ejection fraction and left ventricular volume (27). Furthermore, it enables tissue characterization which can dictate medical management (85). Similar to echocardiography, strain is an emerging biomarker that can help in the ascension of CMR (86, 87). Nevertheless, some processes in CMR take substantial time such as measuring volume or contour tracing (86). The integration of ML architectures can help expand the domain of CMR and transcend into new frontiers in cardiovascular imaging.

Ruijsink et al. presented exceedingly high CNN algorithm correlation with a manual analysis of LV and right ventricular volumes (all *r* > 0.95), strain (circumferential *r* = 0.89, longitudinal *r* > 0.89), and filling and ejection rate (all *r* ≥ 0.93) in CMR (88). Winter et al. showed deep learning achieved a similar or higher performance with human experts for automatic segmentation of the right and left ventricular endocardium and epicardium (89). Bhuva et al. explored automated ML analysis of cardiac structures in CMR, automated analysis was 186 times faster than humans (0.07 vs. 13 min) (90). Jain et al. utilized ML to demonstrate phasic right atrial (RA) phasic is predictive of all-cause death in patients with and without HF, adjusted RA reservoir strain (HR = 0.66, *p* = 0.0154), RA conduit strain (HR = 0.58, *p* = 0.0039), and RA conduit strain rate (HR = 1.51, *p* = 0.0373) independently predicted all-cause mortality (91). Fahmy et al. investigated the role of deep learning in CMR scar quantification for hypertrophic cardiomyopathy, there was strong correlation between automatic and manual segmented scar volumes (*r* = 0.9, *p* < 0.001 per patient and (*r* = 0.84, *p* < 0.001 per slice) and LV mass (*r* = 0.96, *p* < 0.001 per-patient and *r* = 0.93, *p* < 0.001) (92). Neisius applied ML and radiomics to differentiate hypertensive heart disease and hypertrophic cardiomyopathy with native T1 mapping. The selected texture attributes in conjunction with the support vector machine classifier provided maximal diagnostic accuracy (c statistic −0.820) in comparison to T1 mapping (c statistic −0.549) for distinguishing between the two entities (93). Knott et al. studied the role of AI-based quantification of stress myocardial blood flow (MBF) and myocardial perfusion reserve (MPR) in CMR for CAD, reduced quantities of both factors were independently associated with death and MACE (94). Swift et al. utilized ML in CMR to extract features and automated pulmonary artery hypertension (PAH) diagnosis, the AUC of the diagnostic approach was superior to CMR metrics (0.92 for PAH, *p* < 0.001) and less time consuming (95).

## Discussion

### Our Contemporary Views on Artificial Intelligence and Machine Learning

CAD is one of the leading causes of mortality in the world and is responsible for a host of cardiovascular-related complications (59, 96, 97). Although it may be relatively easy to pinpoint the exact cause of death but implementing universal solutions in terms of medications or intervention is not necessarily straightforward. There is a fundamental concept present at hand, it must be greatly emphasized that cardiovascular disease is heterogeneous in nature (98). The pathophysiology of cardiovascular disease encompasses various interactions between etiological factors, molecular components, genetic attributes, and intricate pathways (98). This is further compounded by varying clinical presentations which further complicate diagnosis and prognostication (22). These clinical dilemmas underpin the necessity and integration of ML frameworks in imaging or clinical pipelines in cardiovascular care (13). ML algorithms can extrapolate information from these multi-dimensional matrices to delineate unique patterns not witnessed before (24). Cloud-based infrastructures enhance data collection allowing for individualized care (20). Many studies have already shown the superiority of big data ML algorithms over standard care in areas including heart failure, AS, and ischemic heart disease (Table 2). By harvesting this information routinely, we can customize individualized solutions for medical management. AI can truly usher the era of precision medicine into modern-day medicine (1, 99).

**Table 2:** Recent examples of studies applying machine learning in cardiovascular interpretation.

**Study**	**ML approach**	**Imaging type**	**Brief Study description**
Samad et al. (36)	Supervised learning	Echo	To predict survival by using clinical and echocardiographic data
Zhang et al. (37)	Deep learning	Echo	To achieve automatic interpretation with echocardiographic data
Sanchez-Martinez et al. (38)	Unsupervised learning	Echo	To examine differences between HFpEF and healthy patients
Tabassian et al. (39)	Supervised learning	Echo	To identify patients with HFpEF through spatiotemporal variations of strain during stress and exercise
Mishra et al. (40)	Unsupervised learning	Echo	To identify clusters of HF patients and the risk of HF hospitalization
Przewlocka-Kosmala et al. (41)	Unsupervised learning	Echo	To identify clusters of HFpEF patients
Omar et al. (42)	Unsupervised learning	Echo	To perform cluster analysis of left atrial and left ventricular strain in diastolic dysfunction patients
Salem Omar et al. (43)	Supervised learning	Echo	To characterize left ventricular filling pressure in diastolic dysfunction patients
Lancaster et al. (44)	Unsupervised learning	Echo	To cluster echocardiographic variables to isolate high-risk phenotyping patterns
Asch et al. (45)	ML algorithm	Echo	To examine automatic quantification of ejection fraction
Benjamin et al. (46)	Deep learning	Echo	To examine the relationship between lung Doppler signal with mitral E'/e ratio and outcomes
Kusunose et al. (47)	Deep learning	Echo	To detect wall motion abnormalities
Kusunose et al. (48)	Deep learning	Echo	To automate LVEF
Donal et al. (49)	Supervised learning	Echo	To assess response to cardiac resynchronization therapy
Casaclang-Verzosa et al. (51)	Unsupervised learning	Echo	To identify unique phenotypes during AS progression
Kwak et al. (53)	Unsupervised learning	Echo	To identify which AS clusters are associated with cardiovascular complications
Tokodi et al. (54)	Unsupervised learning	Echo	To detect clusters of patients and predict MACE events
Cho et al. (52)	Unsupervised learning	Echo	To identify clusters of heart failure patients and predict cardiovascular complications
Baskaran et al. (72)	Deep learning	CT	To compare the automatic and manual assessment of left and right heart sided structures and function
Zhou et al. (63)	Multiple ML algorithms	CT	To utilize CT FFR to predict myocardial bridge formation
Oikonomou et al. (73)	Supervised learning	CT	To assess the potential of perivascular fat in cardiac risk prediction
Beecy et al. (74)	Deep learning	CT	To identify acute ischemic stroke in CT
Al'Aref et al. (75)	Supervised learning	CT	To detect culprit coronary lesions in CT for acute coronary syndrome patients
Coenen et al. (64)	Supervised learning	CT	To detect CAD
Kay et al. (70)	ML algorithm	CT	To detect phenotypic information about left ventricular hypertrophy
Eisenberg et al. (76)	Deep learning	CT	To assess the role of epicardial tissue in MACE events
Al'Aref et al. (68)	Multiple ML algorithm	CT	To use coronary calcium and clinical factors to predict CAD
Tesche et al. (69)	ML algorithm	CT	To compare ML CT FFR and CT and CAC
Tang et al. (65)	ML algorithm	CT	To compare ML CT FFR with CTA and invasive angiography
Hou et al. (71)	Supervised learning	CT	To calculate the pre-test probability of CAD
Betancur et al. (78)	Deep learning	Nuclear	To assess CAD prediction
Betancur et al. (79)	Deep learning	Nuclear	To assess CAD prediction in semi-upright and supine stress MPI
Betancur et al. (80)	Supervised learning	Nuclear	To compare MACE predictive accuracy Of ML with expert evaluation
Hu et al. (81)	ML algorithm	Nuclear	To compare rest scan cancellation rates and complications between ML algorithm and physician or clinical systems
Otaki et al. (82)	ML algorithm	Nuclear	To compare MACE predictive accuracy of visual reading with ML networks
Juarez-Orozco et al. (83)	Deep learning	Nuclear	To assess the role of deep learning in polar maps for ischemia
Hu et al. (84)	ML algorithm	CMR	To predict per-vessel prediction of early coronary revascularization in SPECT MPI
Ruijsink et al. (88)	Deep learning	CMR	To compare automatic ventricular measurements with CMR manually
Winther et al. (89)	Deep learning	CMR	To evaluate automatic segmentation of epicardium and endocardium by deep learning
Bhuva et al. (90)	Deep learning	CMR	To evaluate automated analysis
Jain et al. (91)	ML algorithm	CMR	To evaluate right atrial phasic function in predicting all-cause death
Fahmy et al. (92)	Deep learning	CMR	To estimate CMR scar quantification in hypertrophic cardiomyopathy
Neisius et al. (93)	Supervised learning	CMR	To differentiate hypertrophic cardiomyopathy and hypertensive heart disease
Knott et al. (94)	AI algorithm	CMR	AI-based quantification of myocardial blood flow and myocardial perfusion reserve
Swift et al. (95)	Multiple ML algorithm	CMR	To extract features and automate PAH diagnosis

*ML, Machine Learning; AI, Artificial Intelligence; Echo, Echocardiography; CMR, Cardiac Magnetic Resonance; CT, Computed Tomography; CT-FFR, CT Fractional Flow Reserve; MPI, Myocardial Perfusion Imaging; SPECT, single photon emission computed tomography; CAD, Coronary Artery Disease; MACE, major adverse cardiovascular events; PAH, Pulmonary Artery Hypertension; CAC, Coronary Artery Calcium*.

The integration of genomics into ML algorithms will be more beneficial than popular risk scores which are restricted to a few variables. The Framingham Risk Score, for instance, is widely utilized for this purpose but does not have a genetic aspect. Genomics is being increasingly integrated into clinical databases or pan-omic datasets (98). Furthermore, they can provide valuable insight into underlying pathophysiology in various cardiac diseases (23). Among all current approaches, ML algorithms can truly appreciate the depth of information present dormant in these datasets (13, 30). This can lead to the development of new biomarkers or potential drug pathways (22, 23). In the coming days, combing cardiovascular imaging with pan-omic techniques will become the eventual standard in patient care (23). Also, this new information can help better stratify patient populations appropriately (8).

The rise of radiomics will be catapulted by the rise of ML architectures (20). Radiomics allows to extract of more features from imaging in greater detail and facilitates quantitative assessment. These features can be measured and evaluated. They can be particularly advantageous in various heterogeneous conditions. It can also help distinguish between various pathological entities that appear similar to imaging. Furthermore, it can be used to detect certain phenotypes within these conditions (100).

AI is driving the current philosophy in research to evolve and be flexible. The current norm of research is very linear, moving from a hypothesis to a conclusion (14). In reality, our lives and even our pathophysiology are actually multilinear. ML algorithms can analyze data in a variety of manners, we should be able to modify our hypothesis accordingly (23). This allows our research to be very dynamic and this can lead to new data-driven discoveries (14). This mindset may be needed as we move forward with the integration of ML algorithms in cardiovascular imaging.

AI can even improve randomized clinical trials (RCTS) in clinical research and cardiovascular imaging. The findings from RCTS define clinical care and are incorporated into national or international guidelines. Many pivotal RCTs fail to reach enrollment goals or have lofty expectations (101). If AI can analyze preliminary results from clinical trials, investigators can use this information appropriately (102). Better classification of the disease in question will allow the superior design of the RCT, there will be a more precise definition of the underlying condition. ML algorithms can determine which patient profiles may predict response to treatment or susceptibility to complications, this will enable better enrollment strategies (102). The early analysis can be used to redesign the trial or not conduct the trial in the first place which can save resources and time. Implementation of ML algorithms can also augment randomization by introducing additional imaging or clinical attributes (101).

**Figure 4:**
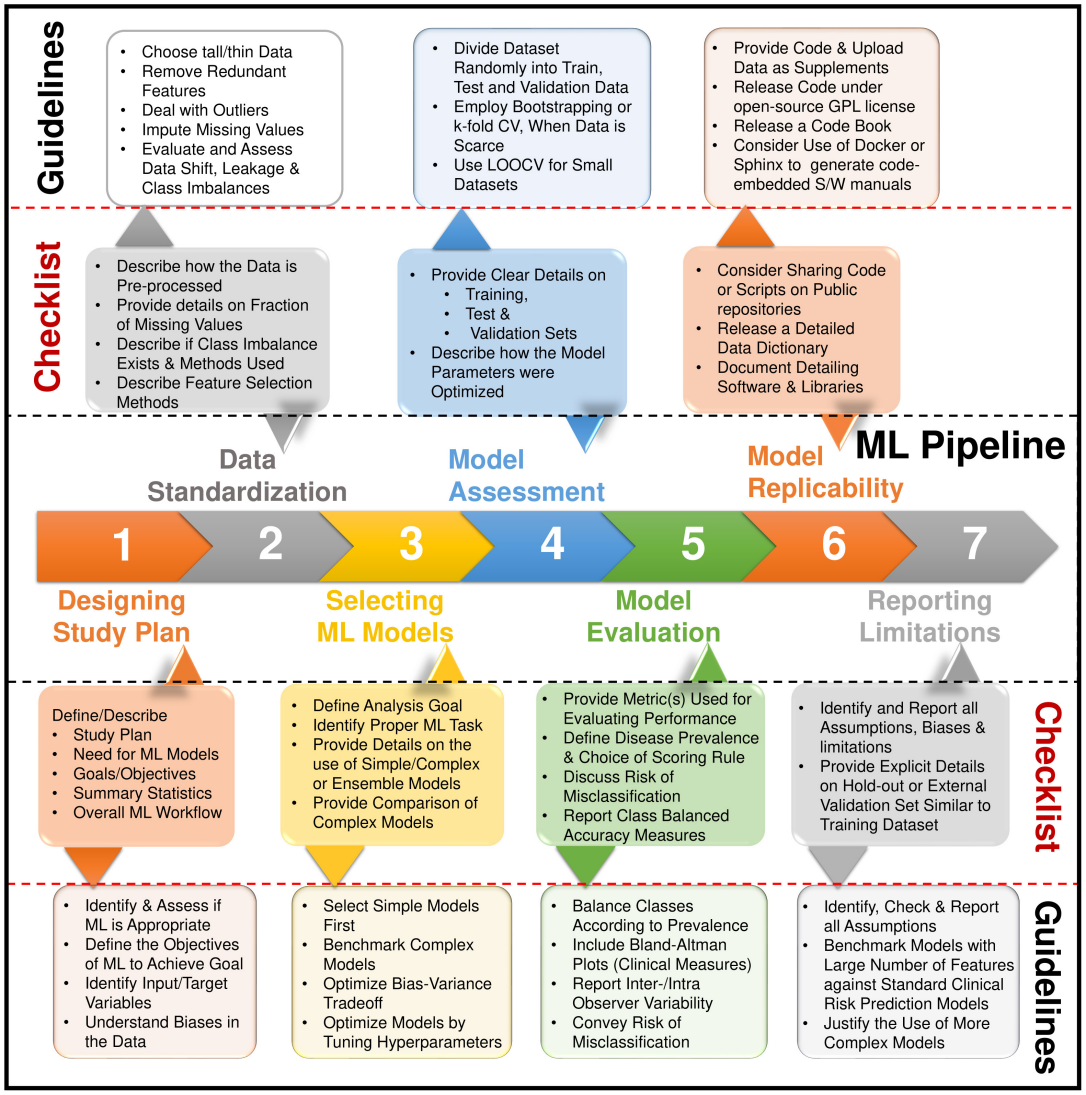
Steps for building a machine learning pipeline and the reporting items in a checklist. Adapted from Sengupta et al. (2).

The benefits of AI and ML algorithms will trickle downwards toward peripheral or community level hospitals. Besides, it can have significant advantages in the imaging lab setting also. AI can automatically develop complex protocols in cardiovascular imaging (Figure 4). Furthermore, it has the capability of adjusting these protocols depending on the situation. It can reduce errors in the acquisition, automate measurements, and greatly improve efficiency (11). This will result in increased decision support and confidence in imaging findings (11). As a result, this can help standardize reporting and improve the overall process. Recent developments are showing the promise of AI in prediction during intervention and management. AI can be used in combination with virtual planning to create digital twins (103). Interventional treatment can be simulated on twin which can plan actual intervention. This can lead to a number of individualized treatment options.

### Potential Issues in Machine Learning

Though the potential of ML algorithms is tremendous, several looming issues need to be addressed for successful integration (12). For any ML algorithm to thrive, it needs adequate exposure to large data sets. This is simpler said than done. Many hurdles need to be overcome before procuring these unique data sets. Institutions must learn to share data among themselves or have some form of agreement in place. Furthermore, multiple institutional review boards (IRBs) are required to share data and it can be a tedious process (17). Besides, data sets need to be de-identified to maintain patient security. If these datasets can be made publicly accessible, then centers can benefit.

Multiple imaging storing systems exist within institutions which include picture archive and communication systems (PACS) or digital imaging and communications in medicine (DICOM). Each academic center may have different protocols in place. A universal data standard needs to be recognized and implemented with minimal variation (104). This will enable the growth of ML in various institutions (104).

The potential for false discovery is a potential pitfall with ML algorithms (12, 25). This can occur with smaller data sets. Investigators need to clearly define the purpose of their research before they interact with ML algorithms. Besides, Investigators need to be constantly vigilant to prevent unintentional biases from creeping into the model. Sampling bias can occur if the data does not capture the heterogeneity of cardiovascular disease. Unintentional prejudicial biases can be introduced into the model. One also must be aware of measurement bias as well. Frequent discussions are needed between the physician and engineers before ML initiation in various research endeavors (18).

The “black box” nature has always been the Achilles heel of ML algorithms and has been a deterrent in its adoption. These algorithms are not programmed to have ethics. For ML algorithms to truly advance in the medical field, physicians need to be properly educated about these topics. Medical school curriculums should introduce ML to medical students, so they have adequate exposure (67). Once they complete their medical training, they can be well-versed and conduct proper research (67).

## Future Directions of Machine Learning

Telemedicine has experienced phenomenal growth in recent years due to miniaturized equipment and wearable devices (3, 105). With the evolution of smartphone applications, this will have a revolutionary impact on medical management (4). With infrastructures, these devices and applications can deliver clinical care to underserved regions throughout the world. We have had positive experiences with handheld ultrasound with cloud technology integration in remote regions of India (106, 107). The data arising from these devices cannot be adequately analyzed by current statistical approaches, it will only be possible with ML algorithms (3).

In parallel with the growth of telemedicine and mhealth, the rapid advances in technology can have a fundamental impact on various healthcare business models. This will lead to the eventual development of “smart” clinics. These clinics usually have an array of miniaturized devices such as pocket ultrasound and smartphone applications. These services will be linked to AI or ML algorithm-driven operations that can analyze information in real-time. This will allow precision medicine to be delivered in each admission or routine follow up. These clinics will be integral to the field of cardiovascular imaging.

## Conclusion

The profound impact of AI in cardiovascular imaging will have monumental effects on clinical care. ML algorithms will connect information from multiple sources in a seamless transition. It will automate several tasks which will provide more time for patient interactions for cardiologists. It will greatly augment the workflow and ultimately improve medical management. AI and ML-driven algorithms are no longer a possibility but an inevitability in the field of cardiovascular imaging.

## Author Contributions

All authors listed have made a substantial, direct and intellectual contribution to the work, and approved it for publication.

## Conflict of Interest

PS was a consultant for HeartSciences and Ultromics. The remaining authors declare that the research was conducted in the absence of any commercial or financial relationships that could be construed as a potential conflict of interest.
